# Hospital discharge readiness based on Meleis’s transition theory and its associated factors among patients after coronary artery bypass grafting: a cross-sectional study

**DOI:** 10.3389/fmed.2026.1718118

**Published:** 2026-05-20

**Authors:** Hayat Abu Shaikha, Maha Atout, Imad Al Jarrah, Eman Alsaleh

**Affiliations:** 1 Department of Nursing, School of Nursing, Al Balqa Applied University, As-Salt, Jordan; 2 Department of Nursing, School of Nursing, Philadelphia University, Amman, Jordan

**Keywords:** CABG, cardiac surgery, discharge, education, readiness, sociodemographic

## Abstract

**Introduction:**

Readiness for hospital discharge is an ongoing concern for healthcare providers. There is currently limited evidence on patients’ readiness for hospital discharge after coronary artery bypass grafting (CABG) among the Jordanian population. This study aimed to assess the readiness for hospital discharge levels among patients post-CABG surgery and to identify its associated factors in a large cardiovascular center in Jordan using the transition theory by Meleis.

**Method:**

A cross-sectional design was used across a total of 203 patients who were selected using a convenience sampling procedure. The Readiness for Hospital Discharge Scale (RHDS) was used to collect data on the day of hospital discharge. Statistical analysis, including the chi-square test and multiple linear regression tests, was then used to identify factors associated with readiness for hospital discharge.

**Results:**

The total mean readiness scale score was 6.69 (±1.311), indicating a moderate level of discharge readiness. Patients in this study scored highest on physical readiness (*M* = 7.61, SD = 1.568) and availability of help for medical care (*M* = 7.76, SD = 1.737); however, lower scores emerged regarding knowledge of potential post-discharge problems (*M* = 5.02, SD = 2.290) and knowledge regarding restrictions after discharge (*M* = 5.26, SD = 2.328). Illiteracy (*B* = −0.740, *p* = 0.001), education lower than secondary level (*B* = −0.635, *p* = 0.010), and older age (*B* = −0.021, *p* = 0.021) were significantly associated factors with lower readiness for hospital discharge.

**Conclusion:**

Healthcare providers should seek to assess readiness for discharge among post-CABG patients comprehensively to offer special education and support to those patients at highest risk to enhance their recovery post-CABG surgery.

## 1 Introduction

Coronary artery diseases (CADs) are the primary cause of death worldwide, and they specifically denote the coronary atherosclerotic disease of the coronary arteries that supply the heart (1). It is a significant type of cardiovascular disease (CVD) that encompasses a number of conditions that include ischemic heart disease (IHD/CAD), stroke, peripheral artery disease, hypertensive heart disease, rheumatic heart disease, cardiomyopathy, myocarditis, valvular disease, atrial fibrillation, endocarditis, and other circulatory diseases. In Jordan, CVD more generally is the leading cause of death, constituting 42% of all deaths in the country (2). The prevalence of CVD in Jordan also increased markedly, by 22%, between 1990 and 2019, with cases rising from 121,232 in 1990 to 546,588 in 2019 (3). Coronary artery bypass grafting (CABG surgery) is a key revascularization strategy recommended by contemporary guidelines for severe CAD (4) that helps restore adequate blood flow, relieves chest pain, improves heart muscle function, and improves survival (5).

The recovery process after CABG surgery can be extensive, involving challenges for nurses and caregivers (6). A patient who has undergone CABG surgery has the potential risk of many post-surgery complications (7). Such complications might include prolonged hospitalization times, anxiety, delirium, bleeding, cardiac tamponade, respiratory complications, wound infections, and strokes (8). Recovery from CABG can also be further extended or hampered by a range of both physical and psychological difficulties (6), causing many patients post-CABG surgery to experience heightened vulnerability during the transition from hospital to home.

Hospital discharge planning is an important part of safe hospital-to-home transitions and has been identified as an extremely vital process to reduce complications, enhance treatment compliance, and avoid hospital readmissions. Proper discharge planning will make sure that patients have been taught about medications, activity restrictions, symptom management, and post-discharge needs and help in continuity of care following hospitalization.

These considerations are particularly important for patients undergoing significant cardiac surgery, such as coronary artery bypass graft (CABG) surgery. Accurate assessment of readiness for hospital discharge among CABG patients helps healthcare providers to ensure that patients are physically and mentally prepared to manage a range of different risks, such as wound infection, arrhythmia, and adherence to medications and activity restrictions. It also supports safe self-management at home by helping to determine whether the patient understands their medication regimen, can care for themselves, knows when and how to seek help, and is aware of signs of complications; this is particularly very important in Jordan, where many patients must return home to rural areas that have limited access to immediate cardiac healthcare (9). Additionally, patients in Jordan have widely varying levels of health literacy (10). Within the Jordanian culture, family involvement is a vital element in patient care, and assessing readiness for hospital discharge also allows more effective involvement of family care providers in discharge planning by providing healthcare staff with information regarding the aspects of training and information that need to be provided to family caregivers to ensure a supportive home environment (11). Developing a discharge plan for a patient after open-heart surgery can thus effectively contribute to reducing expected complications after the operation, thereby both alleviating the patient’s suffering and reducing the patient’s length of stay (LOS) in the hospital (8). Effective discharge planning must therefore encompass recovery expectations, complication prevention, and self-care instructions (12). In Jordan, patients undergoing CABG are typically in their late 50s; in a recent single-center study, the median age was 58.5 years (13). This is comparable to reports from other Middle Eastern countries, where mean ages of CABG patients are approximately 58–63 years (14), and somewhat younger than cohorts from Europe and North America, where mean ages are often in the mid-60s (15). These data highlight that CABG is frequently performed in relatively older adults, underscoring the importance of readiness for discharge and postoperative support in this age group.

The evaluation of a patient’s readiness for hospital discharge has been widely recognized as a fundamental element of the discharge planning process (16, 17). Numerous observational studies have also shown that inadequate patient readiness for hospital discharge, as evaluated by nurses and patients on the day of discharge, correlates significantly with challenges in coping post-discharge and an elevated probability of the patient returning to the hospital for an emergency department visit or inpatient readmission within 30 days of discharge (18–21). In one study, evaluations by nurses indicating low discharge readiness correlated with a sixfold to ninefold elevation in readmission risk for general medical and surgical patients (22).

Patients undergoing CABG experience one of the highest readmission rates among those undertaking major surgeries, with meta-analysis revealing a 12.9% 30-day readmission rate (23). Moreover, over half of these readmissions (53%) are attributable to non-cardiac causes, such as respiratory infections, sepsis, and wound complications (23, 24). In a Brazilian study, 63% of readmissions were for non-cardiovascular conditions, most notably surgical-site infections (20.5%) and pneumonia (10.3%) (25).

Unreadiness for hospital discharge was also found to be a predictor of increased readmission risk within 1 year of discharge among medical patients with cardiac diseases (26). Rotvig and Christensen (1) have indicated that four attributes were markedly compromised in patients who felt unprepared for hospital release: physical stability, adequate support, psychological ability, and adequate information and knowledge. The study by Rotvig and Christensen has shown that patients who lived alone, were divorced, were less adherent to their medications, and/or had chronic disorders commonly reported feeling unready for discharge, as did patients who felt that their condition was uncontrolled. Those who reported feeling unprepared expressed a particular need for information on the health concerns that needed to be monitored and who to contact for post-discharge treatment (26).

To provide the required support and necessary interventions to patient’s post-CABG surgery, it is important to strive to understand their situation at the time of discharge fully. Current knowledge about patients’ readiness for hospital discharge post-CABG surgery is incomplete; however, the previous literature in this area has concentrated on general medical surgical patients, with no emphasis on specialized major surgeries. Moreover, there is even more limited knowledge regarding discharge preparedness among CABG patients in Jordan, as current research rarely considers the distinctive cultural and healthcare setting of this population. The current research, informed by transition theory (27, 28), thus aimed to evaluate preparedness for hospital discharge among Jordanian patients post-CABG surgery, examining readiness for hospital discharge levels among cardiac patients post-CABG surgery and thereby identifying the major associated factors, including age, gender, education level, marital status, occupational status, smoking history, chronic diseases, household members, date of diagnosis, date of discharge, length of stay at intensive care unit, and also the length of stay at a hospital.

Although hospital discharge readiness has been a common practice and a topic in clinical practice and research, it is a theoretically ambiguous concept that has been inconsistently defined. Concept analyses also point to a lack of an operational definition as comprehensive, which adds to differences in conceptualizing and measuring discharge readiness in different ways. The Readiness for Hospital Discharge Scale (RHDS) has been modified and tested with older adults (RHDS-OP and RHDS-OP short form) and has become common in the geriatric and post-acute settings and elderly patients after major cardiac and orthopedic surgery, which validates its appropriateness in the mostly middle-aged and older patient’s post-CABG in this study (29). Systematic and scoping reviews have also suggested that current measures focus on specific areas of discharge preparedness, including physical condition, psychological state, knowledge, coping capacity, and expectations of support, thus restricting conceptual consistency across studies. To resolve this multidimensional complexity, the RHDS was created, in which the discharge readiness was arranged into four central domains: personal status, knowledge, coping ability, and expected support. Such areas are especially pertinent to the patients who will undergo coronary artery bypass graft (CABG) surgery and cope with polypharmacy, wound and symptom care, lifestyle changes, and the use of post-discharge support systems. Nonetheless, the RHDS implementation in post-CABG cohorts should be done with great caution since varying recovery pathways and transitions to care can affect perceptions and readiness assessments of patients.

### 1.1 Theoretical framework

This study was also informed by the transition theory by Meleis, which describes the process of experiencing and adapting to health-related transitions by the individual (27, 28). The hospital-to-home CABG postoperative transition is a vulnerable and acclimatization phase that patients face, and, as per the theory, effective transitions are conditioned by the state of personal and environmental factors, as well as society, and are characterized by the symptoms of awareness, engagement, and mastery. The Readiness for Hospital Discharge Scale (RHDS) (30, 31) measures five key dimensions—physical, psychological, education and knowledge, adequate individual support, and social and organizational determinants—that align closely with core concepts of transition theory such as awareness, engagement, coping, and support. The education and knowledge dimension corresponds to awareness, reflecting patients’ understanding of their condition and care needs necessary for a successful transition. The psychological dimension relates to engagement and coping, capturing emotional readiness and confidence in managing health demands after discharge. The adequate individual support dimension directly reflects the concept of support, emphasizing the availability of help from family, healthcare providers, or community resources during the transition. Physical readiness addresses the patient’s functional status, which influences their ability to cope with daily activities post-discharge. Social and organizational determinants highlight external factors that can affect all these core concepts by either facilitating or hindering a smooth transition. Together, these RHDS dimensions provide a comprehensive framework to assess how well patients are prepared across the critical elements of transition theory for moving safely from hospital to home.

### 1.2 Research questions

The specific research questions were as follows:

- What is the level of readiness for hospital discharge among Jordanian cardiac patients post-CABG surgery?
- What are the factors associated with levels of Jordanian cardiac patients’ readiness for hospital discharge post-CABG surgery?

## 2 Materials and methods

### 2.1 Design

As discharge readiness is a time-bound construct and routinely assessed within a few hours before a patient is discharged from the hospital, a cross-sectional design was used. Moreover, this design was selected as it is among the most effective at identifying patterns or relationships, based on its use to observe and describe aspects of a situation as it naturally occurs (32). Additionally, it allows efficient inclusion of multiple variables in multivariable analyses. Moreover, this design is useful for identifying associations and generating hypotheses about potential relationships without requiring long follow-up periods (33). It can serve as a foundation for future longitudinal research by highlighting key associated factors that warrant further research.

### 2.2 Setting

This research was performed at a prominent military hospital for heart surgery in Amman, Jordan. The hospital was selected due to its extensive provision of cardiology and cardiac surgical procedures for a diverse patient group with a range of cardiovascular problems. Moreover, restricting recruitment to one institution ensured a consistent surgical procedure and discharge process among all patients and reduced variability related to differing institutional policies. In Jordan, discharge planning is initiated by the bedside nurse once the medical team has decided on discharge. Routine discharge education is typically delivered on the day of discharge and focuses on verbal instructions about diagnosis, prescribed medications, wound care (if relevant), activity level, diet, and follow-up appointments; written discharge summaries and medication prescriptions provided to the patient and/or family; and a brief opportunity for questions. Formal, structured discharge planning models and standardized discharge teaching tools are not yet systematically implemented, and there is no routine, standardized assessment of readiness for hospital discharge. Discharge resources are largely limited to outpatient clinic follow-up and telephone contact with the hospital; community-based or home-care services are not consistently available. In addition, education is typically provided to both patients and family members, reflecting the important caregiving role of families in the Jordanian context. This education usually includes instructions on medication management, wound care, activity restrictions, dietary advice, recognition of warning signs, and scheduling follow-up appointments. However, structured written educational booklets, standardized discharge teaching protocols, and formal caregiver training sessions are not consistently implemented across patients. The study was conducted from September 2024 to January 2025, with all cardiac patients who received CABG surgery within that period invited to participate.

### 2.3 Sample and sampling

In this research, a convenience sample was used. The rationale behind this approach was feasibility, and this is an approach used in other studies that measure discharge readiness in cases of hospital discharge. The head nurses in three cardiac surgery wards (male floor, female floor, and the intermediate ICU) were approached every day to invite the patients who were scheduled for discharge to participate in the study. All the patients who were scheduled for discharge according to their surgeon were approached to participate. By approaching the three floors, we tried to minimize the sampling bias that might be associated with the convenience sampling procedure. Moreover, we implemented strict inclusion criteria to further minimize the sampling bias. We selected patients who were (1) adults (18 years and above), (2) had undergone an initial isolated elective CABG surgery to treat single, double, or triple coronary artery disease, and (3) spoke Arabic and were willing to participate and able to make an informed consent. Prior to the sample size calculation, the calculation was performed using G*Power software (F tests, linear multiple regression: fixed model). Given a medium effect size (*f*^2^ = 0.15), *α* = 0.05, power = 0.95, and 7 predictors, the minimum required sample size was 153 participants. To consider the incomplete responses, 225 questionnaires were distributed, and 203 full responses were used in the final analysis.

### 2.4 Instruments

#### 2.4.1 Personal information form

The personal information form was ready for the researchers based on detailed literature analysis. The form was validated by the research team and piloted with the first 10 participants in the study. No revisions were required. The form therefore contained 13 questions on sociodemographic and clinical health concerns. The data gathered included the following: age, gender, education level, marital status, occupational status, smoking history, chronic diseases, household members, date of diagnosis, date of discharge, length of stay at the intensive care unit, and length of stay at a hospital. All these data were collected from the patients’ files.

#### 2.4.2 Readiness for hospital discharge scale

- The Readiness for Hospital Discharge Scale assesses a patient’s preparedness to return home after acute care hospitalization. The RHDS/SF uses a scale of 0 to 10, focusing on eight elements across four dimensions. An RHDS/SF score of ≥7 indicates that the patient is ready for discharge, whereas a score of <7 indicates that the patient is not prepared to leave the hospital. The cutoff score of ≥7 on the Readiness for Hospital Discharge Scale-Short Form (RHDS/SF) is based on prior validation studies demonstrating its clinical relevance. Research has shown that nurse assessments using a threshold below 7 are associated with a significantly increased risk of 30-day hospital readmission, indicating that patients scoring ≥7 are generally considered ready for discharge (19) This threshold has been supported by psychometric evaluations confirming the scale’s reliability and predictive validity in medical-surgical populations (19, 22). Additionally, versions of the RHDS/SF have been validated in different cultural contexts, such as the Turkish population, showing consistent reliability and construct validity around similar scoring ranges (18). While some adaptations exist for specific patient groups or settings, the cutoff score of ≥7 is widely accepted in adult discharge readiness assessment literature and aligns with established clinical practice (34–36). The four dimensions assessed are as follows:
- Personal status, which refers to the patient’s physical and emotional condition at the time of discharge.
- Knowledge, which assesses whether the patient has the information required to address post-discharge complications.
- Coping ability, which refers to the patient’s capacity to manage their personal and healthcare needs independently at home.
- Expected support denotes the extent of assistance and emotional support available to the patient (30, 37).

The initial English version has been translated into Arabic, and it is published on the official site of the instrument developer university webpage.^1^ This version has been confirmed as valid and reliable (31). In this study, the RHDS/SF showed strong internal consistency and reliability, with a Cronbach’s alpha of 0.83.

### 2.5 Data collection procedures

After obtaining the required permissions from the hospital and the potential participants in the study, the head nurses and the charge nurses on the surgical floors were informed about the purpose of the study and the procedures of data collection to allow them to facilitate the work of the researchers. The nurses responsible for the target patients were contacted daily to identify those patients about to be discharged. All the patients who were scheduled for discharge met the discharge criteria according to the cardiac surgeon responsible for their care. All these patients were approached and invited to participate in the study. The participants were fully informed about the study and the voluntary nature of their participation in advance and then were approached by the researcher 4 h before their discharge from the hospital to complete the instrument. The timing of data collection 4 h before hospital discharge may indeed introduce response bias due to patient fatigue, stress, or anxiety at that moment. To minimize this bias, a few measures were taken, such as ensuring a calm environment by meeting the patients in their rooms, answering their questions while completing the scale, and allowing them sufficient time to respond. The questionnaire required approximately 15 to 20 min for completion. To take into account the standard dropout rate of 5 to 10% seen in many cross-sectional studies, 225 questionnaires were distributed to potential participants. A total of 203 questionnaires were returned, giving a response rate of 90.2%. Of these, 22 questionnaires were excluded due to incompleteness.

### 2.6 Ethical considerations

Ethical approval for this study was secured from the hospital’s Ethics Committee under the number 10/2024. Participation was optional at all stages. A short interview was conducted with the head nurse supervisors to familiarize them with the study and the questionnaire, allowing them to more effectively assist with the identification of patients scheduled for discharge. An information sheet was provided to each participant who consented to take part, and all participants were assured that their information would be anonymized through the use of numerical identifiers and codes before being stored on the researcher’s personal computer in a confidential file protected by a password. All participants thereby provided informed, signed consent.

### 2.7 Data analysis

The statistical analysis for this study was performed using IBM SPSS version 27. Continuous variables were summarized as means with standard deviations (SD), and categorical variables were expressed as frequencies and percentages. The normality was assessed using Shapiro–Wilk or Kolmogorov–Smirnov tests. As the data were non-normal, the Mann–Whitney U-test was used for comparisons between two independent groups, and the chi-square test was used for comparisons between categorical variables.

Before linear regression analyses were performed, several assumptions were evaluated, including linearity (using scatterplots), independence of residuals (Durbin–Watson test), homoscedasticity (by visual inspection of residual plots), and normality of residuals (using Q–Q plots). The regression model included variables based on the theoretical framework of the study and prior empirical studies. Using the transition theory developed by Meleis, the factors that indicated personal and health-related transition conditions were deemed to be significant in discharge readiness, including age, gender, education level, marital status, employment status, family size, smoking status, length of hospital stay, and cardiometabolic comorbidity. Multicollinearity was assessed using tolerance and variance inflation factor values. All statistical tests were two-tailed, with a *p*-value of <0.05 considered statistically significant.

## 3 Results

### 3.1 Patient demographic and clinical characteristics

Sociodemographic and clinical characteristics of the study sample are presented in Table 1. The sample consisted of 203 patients, with a mean age of 57.56 years (SD = 10.94, range: 19–85). The majority of participants were male (*n* = 167, 82.3%) patients and were married (*n* = 192, 94.6%). Illiteracy was reported in 42.9% (*n* = 87) of the participants, 29.1% (*n* = 59) had completed secondary education or less, and 28.1% (*n* = 57) were university students or graduates. The average length of hospital stay was 11.25 days (SD = 7.95, range: 4–55), and more than half of the participants (55.2%, *n* = 112) were unemployed.

**Table 1:** Sociodemographic and clinical characteristics of patients (*N* = 203).

Continuous variables (mean ± SD, range)	
Variable	Mean ± SD (Range)
Age (years)	57.56 ± 10.94 (19–85)
Hospital length of stay (days)	11.25 ± 7.95 (4–55)

Categorical variables (*n*, %)			
Variable	Category	*n*	%
Sex	Male	167	82.3
	Female	36	17.7
Education	Illiterate	87	42.9
	Secondary or lower	59	29.1
	University	57	28.1
Marital status	Single	7	3.4
	Married	192	94.6
	Other	4	2.0
Employment status	Unemployed	112	55.2
	Employed	91	44.8
Diabetes	Yes	104	51.2
	No	99	48.8
Hypertension (BP)	Yes	112	55.2
	No	91	44.8
Triglycerides (TG)	Elevated	68	33.5
	Normal	135	66.5
Kidney disease	Yes	1	0.5
	No	202	99.5
Smoking status	Yes	29	14.3
	No	174	85.7

Hypertension (*n* = 112, 55.2%) and diabetes (*n* = 104, 51.2%) were the most prevalent clinical conditions. In contrast, elevated triglycerides (*n* = 68, 33.5%) and smoking (*n* = 29, 14.3%) were less common. Although the sample showed a high incidence of metabolic conditions, renal complications were rare, with kidney disease reported in only 0.5% of patients.

### 3.2 Patient readiness for hospital discharge

Self-assessment of patient readiness for hospital discharge was evaluated across several dimensions (Table 2). Patients reported the highest scores with respect to physical readiness (*M* = 7.61, SD = 1.568) and the availability of help regarding medical care (*M* = 7.76, SD = 1.737). Availability of personal care assistance (*M* = 7.55, SD = 1.960) and energy levels (*M* = 7.33, SD = 1.713) were also rated quite well. In contrast, lower scores were reported for knowledge of post-discharge problems (*M* = 5.02, SD = 2.290) and awareness of activity restrictions (*M* = 5.26, SD = 2.328). Moderate scores were also observed regarding patients’ ability to handle home demands (*M* = 6.16, SD = 1.812) and performance of personal care (*M* = 6.89, SD = 1.917). The mean overall readiness score was 6.69 (SD = 1.311), indicating a moderate level of discharge readiness.

**Table 2:** Hospital discharge readiness assessment (*n* = 203).

Question	Mean score	Standard deviation
Physical readiness	7.61	1.568
Energy level	7.33	1.713
Knowledge of post-discharge problems	5.02	2.290
Knowledge of restrictions	5.26	2.328
Ability to handle home demands	6.16	1.812
Ability to perform personal care	6.89	1.917
Help available for personal care	7.55	1.960
Help available for medical care	7.76	1.737
Overall readiness for hospital discharge	6.69	1.311

### 3.3 Patient readiness for hospital discharge and demographic and clinical characteristics

Readiness for discharge was then analyzed in relation to significant demographic and clinical variables using chi-square analyses (Table 3). According to the RHDS cutoff score, more than half of the patients were classified as unready for discharge from the hospital (57.1%, *n* = 116), while 42.9% (*n* = 87) were classified as ready. Based on this, readiness was not significantly associated with gender, *χ*^2^ (1, *N* = 203) = 0.281, *p* = 0.596. Similarly, no significant differences in preparedness were found based on work status, *χ*^2^ (1, *N* = 203) = 1.03, *p* = 0.254; diabetes status, *χ*^2^ (1, *N* = 203) = 1.03, *p* = 0.311; blood pressure, *χ*^2^ (1, *N* = 203) = 1.30, *p* = 0.254; or smoking status, *χ*^2^ (1, *N* = 203) = 0.406, *p* = 0.524. Marital status was also not significantly associated with discharge readiness, *χ*^2^ (2, *N* = 203) = 2.89, *p* = 0.236. However, a statistically significant association was found between education level and readiness, *χ*^2^ (2, *N* = 202) = 17.62, *p* < 0.001, indicating that higher education was associated with greater readiness. Additionally, and interestingly, participants with elevated triglyceride levels reported significantly higher readiness; however, this finding should be interpreted cautiously given its unexpected direction and the possibility of residual confounding, *χ*^2^ (1, *N* = 203) = 7.08, *p* = 0.008. Overall, education level and triglyceride level were thus significantly associated with discharge readiness, while other sociodemographic and health-related variables were not associated with discharge readiness. Since several bivariate tests were performed, the chi-square results may be viewed as a form of exploratory analysis, with a potential type I error inflation.

**Table 3:** Patients’ readiness for discharge with respect to their sociodemographic and clinical characteristics.

Characteristics	Unready	Ready	Test statistics	*p*-value
Age				
Sex			*X*^2^(1) = 0.701	0.402
Female	92 (63.9)	52 (36.1)		
Male	72 (56.3)	56 (43.7)		
Education			*X*^2^(1) = 18.938	<0.001
Illiterate	33 (73.3)	12 (26.7)		
Secondary or lower	90 (54.2)	76 (45.8)		
University	42 (36.8)	72 (63.2)		
Marital status			*X*^2^(1) = 2.954	0.228
Single	2 (28.6)	5 (71.4)		
Married	70 (58.3)	50 (41.7)		
Other	66 (75.0)	22 (25.0)		
Work status			*X*^2^(1) = 1.029	0.31
No	66 (54.5)	55 (45.5)		
Yes	56 (61.5)	35 (38.5)		
Diabetes			*X*^2^(1) = 1.330	0.249
No	83 (53.5)	72 (46.5)		
Yes	45 (61.5)	28 (38.5)		
Blood pressure			*X*^2^(1) = 1.029	0.31
No	56 (61.5)	35 (38.5)		
Yes	66 (54.5)	55 (45.5)		
Triglycerides			*X*^2^(1) = 7.652	0.006
No	76 (64.4)	42 (35.6)		
Yes	52 (44.1)	66 (55.9)		
Smoking			*X*^2^(1) = 0.484	0.487
No	82 (58.6)	58 (41.4)		
Yes	46 (51.7)	43 (48.3)		

### 3.4 Associated factors of readiness for discharge

To examine the factors influencing patients’ readiness for hospital discharge in detail, a multiple linear regression analysis was conducted (Table 4). The overall model was statistically significant, *F* (10, 191) = 3.38, *p* < 0.001, accounting for 15% of the variance in discharge readiness (*R*^2^ = 0.15; adjusted *R*^2^ = 0.106). No evidence of problematic multicollinearity was observed among independent variables, with all tolerance values >0.1 and variance inflation values <5.

**Table 4:** Linear regression analysis predicting readiness for hospital discharge.

Predictor	B	SE	*β*	*t*	*p*	95% CI for B
(Constant)	8.330	0.903	–	9.227	<0.001	[6.549, 10.111]
Gender	−0.291	0.253	−0.084	−1.151	0.251	[−0.789, 0.207]
Age	−0.021	0.009	−0.189	−2.319	0.021	[−0.040, −0.003]
Work status	−0.326	0.193	−0.124	−1.687	0.093	[−0.706, 0.055]
Smoking status	0.229	0.264	0.061	0.869	0.386	[−0.291, 0.750]
Family number	−0.031	0.045	−0.054	−0.686	0.493	[−0.120, 0.058]
Length of stay (LOS)	0.001	0.012	0.003	0.048	0.962	[−0.022, 0.023]
Education (illiterate)	−0.740	0.229	−0.279	−3.233	0.001	[−1.192, −0.289]
Education (secondary/lower)	−0.635	0.245	−0.220	−2.590	0.010	[−1.119, −0.151]
Marital status (single)	0.584	0.882	0.081	0.662	0.509	[−1.156, 2.324]
Marital status (married)	0.183	0.645	0.032	0.284	0.777	[−1.089, 1.454]
Chronic disease	0.302	0.254	0.087	1.188	0.236	[−0.199, 0.803]

*R*^2^ = 0.150, adjusted *R*^2^ = 0.101, *F* (11, 190) = 3.043, *p* = 0.001. Dependent variable: readiness for hospital discharge.

Education level was significantly associated with readiness for discharge. Patients who were illiterate or had less than a secondary education showed significantly lower readiness scores than those with a university education [*B* = −0.77, 95% CI (−1.22, −0.33), *p* < 0.001; *B* = −0.67, 95% CI (−1.15, −0.19), *p* = 0.007, respectively].

Age was another significant factor associated with readiness for hospital discharge, with older patients showing lower readiness scores (*B* = −0.027, *p* = 0.007). Interestingly, the length of hospital stay was not significantly associated with readiness (*B* = 0.001, *p* = 0.967). Other variables, such as gender, employment status, smoking status, family size, marital status, and the presence of cardiometabolic comorbidity, did not significantly predict discharge readiness (all *p* > 0 0.05). Although triglyceride status showed a significant unadjusted association with discharge readiness, this finding did not persist in the adjusted analysis. Therefore, it should be interpreted cautiously and considered hypothesis-generating rather than conclusive.

## 4 Discussion

This study examined readiness for hospital discharge among patients following coronary artery bypass graft (CABG) surgery and identified several important findings. Overall, patients showed a moderate level of discharge readiness, suggesting that although many felt physically prepared to leave the hospital, gaps remained in other areas of preparedness. In particular, knowledge-related domains showed lower scores, especially regarding post-discharge complications and activity restrictions. In addition, age and education level were identified as significant predictors of discharge readiness, with older patients and those with lower educational attainment reporting lower readiness scores.

These findings were consistent with previous studies conducted among patients with acute coronary syndrome in China (12). Higher scores in physical readiness and availability of support suggest that patients generally felt medically stable and socially supported at discharge. These results suggest that most patients perceived themselves to be physically stable and well assisted by the HCPs who offered help to them. This reflects the sound quality of medical care regarding physical aspects of the discharge plan during such patients’ hospitalization. Moreover, the high scores for the personal care assistance and availability of help with medical care domains may reflect supportive aspects of Jordanian culture, where family members are commonly involved in patients’ care and decision-making during illness and recovery (2). The moderate level of readiness observed in this study suggests that although patients felt physically stable at discharge, important gaps remained in their preparedness for self-management at home, including the need to equip both patients and their family caregivers with culturally appropriate education, skills, and resources to support ongoing care in this highly family-involved context. The family history was not systematically evaluated as a part of the structured data collection tool applied in this study and, thus, could not be included in the statistical analysis; nevertheless, it might be a clinically relevant factor that affects recovery expectations and discharge readiness and, therefore, should be considered in the future. Moderate readiness scores regarding the ability to handle home demands (M = 6.16, SD = 1.812) and perform personal care (M = 6.89, SD = 1.917) suggest a need for improvement with respect to self-management plans and behavioral changes. However, no documented scores for specific readiness items for discharge have been recorded in previous research, so comparisons are not possible at this time. Documenting items of readiness for discharge is thus essential for future researchers to understand the unique needs of patients being assessed for their readiness for discharge and thus to address these needs in the resulting discharge plans or structured discharge programs. Significantly, this result adds new context-specific information in Jordan, where structured discharge-readiness evaluation is not commonly applied in cardiac surgical hospitals and where patients tend to count on family members instead of obtaining formal post-discharge support services. Considering the growing role of cardiovascular disease in Jordan (3), a gap in knowledge-based preparedness in CABG patients can be viewed as a promising field of advancement related to the practice of discharge education in the local healthcare setting.

There is also the possibility that certain patients may have prior knowledge about their condition and postoperative care due to prior visits to the outpatient clinic or prior encounters with healthcare professionals before surgery, which might have affected their perceived readiness to leave the hospital.

The lowest scores were for knowledge of post-discharge problems (*M* = 5.02, SD = 2.290) and awareness of activity restrictions (*M* = 5.26, SD = 2.328), which underlines potential limitations in the provision of knowledge and education by staff who care for patients post-CABG surgery. Previous studies have suggested that discharge education practices and nursing experience may influence patients’ readiness for discharge (38); however, these factors were not directly assessed in this study and should therefore be interpreted cautiously. A nurse’s role in caring for patient’s post-CABG must include evaluating patient readiness to cope with their new condition in their home environment to improve their ability to care for themselves and modify their behaviors and lifestyle patterns. This is vital, as a patient’s knowledge of required behavioral changes in the post-discharge period is a key factor in lowering the incidence of disease recurrence and improving the patient’s prognosis after coronary artery disease (39). A patient’s adoption of healthy practices post-discharge could thereby enhance their functioning and mitigate the adverse impacts of the disease (40).

The current findings are consistent with Meleis’s transition theory (28), confirming that successful transitions rely on patient preparation, knowledge, and contextual support. Insufficient preparation in elderly and less-educated patients may indicate a compromised or inadequately supported transition phase. However, it is essential to investigate the teaching effect further, examining it as the primary dimension in transition theory with respect to readiness for discharge among such patients in more studies. This study, in contrast, aimed primarily to identify sociodemographic factors that could influence discharge planning, including patients’ readiness for discharge after CABG.

The age factor was also found to be an essential variable in this study, as older patients showed lower readiness for discharge than younger patients, which is consistent with previous research among cardiac populations (41) and may be explained in several ways. Older patients showed lower readiness for discharge than younger patients, which is consistent with previous research among cardiac populations (36). Although this study did not directly assess factors such as comorbidities, anxiety levels, or financial constraints, previous literature suggests that these variables may influence older patients’ ability to manage postoperative recovery and discharge requirements (42). Therefore, these explanations should be interpreted as possible contributing factors rather than conclusions derived from this study.

Additionally, elderly patients might have higher illiteracy rates or lower educational levels that might hinder their understanding or comprehension of instructions post-surgery, which would affect their levels of readiness for hospital discharge. Finally, elderly patients might experience higher levels of anxiety at the time of discharge due to a lack of confidence in their abilities to care for themselves at home; similarly, they might feel the pressure of additional financial constraints that might limit their ability to seek appropriate healthcare when needed (42).

Illiteracy is another significant factor that affects patients’ readiness for discharge. Patients who were illiterate or had less than a secondary education showed lower readiness scores than those with a university education, consistent with previous research among heart failure patients (41, 43). In contrast, providing adequate discharge information prior to early discharge was considered a significant factor that assisted patients’ transition from hospital to home (44). This indicates the need for nurses and healthcare providers to promote discharge planning by taking into consideration high-risk patients’ needs, based on their sociodemographic factors, to prepare patients for discharge readiness during their stay in the hospital by meeting those needs. The result that education level was a significant factor in determining discharge readiness underscores the need to tailor discharge teaching to the health literacy levels of patients. Healthcare providers ought to think about employing simplified educational materials, visual instructions, teach-back techniques, and repeated reinforcement of key information to improve patients’ understanding of post-discharge care. Engaging family caregivers in educational activities can also help to assist patients with lesser educational levels and provide them with more skills in managing recovery safely upon discharge.

Surprisingly, the results of this research revealed that the participants with higher levels of triglyceride levels reported increased discharge readiness. It is a counterintuitive finding clinically, since higher triglyceride levels are usually related to worse cardiovascular risk profiles. The first one is that there might be some confounding factors that have not been measured or some sample-specific factors instead of a clinical relationship. Thus, this result must be viewed with some reserve, and it must be confirmed in future research. This correlation can also be a residual confounding factor, variation in clinical features or recovery patterns of patients, or a statistical artifact due to the sample distribution as opposed to a clinical association.

Nevertheless, although other sociodemographic variables such as marital status, household income, and psychological support were reported in other studies as factors affecting readiness to be discharged from hospitals (41, 45) in this study, there were no significant associations between the readiness to discharge and the aforementioned factors. This observation can be partly attributed to the fact that the family support systems in the Jordanian environment are very strong and that family members often offer physical, financial, and moral support to the patient following surgery. In the same manner, discharge readiness was not significantly associated with gender, smoking status, and employment status in this study. The variations in findings may be due to the differences in sample characteristics, settings of healthcare, and discharge education practices among studies.

In line with the Meleis’s transition theory (28), age and education are significant personal transition conditions that determine the readiness of patients to discharge. Reduced preparedness in old and less-educated patients can indicate variations in knowledge, coping ability, and confidence in the recovery. The reduced knowledge-related preparedness in this study also indicates that a number of patients might be in a very early stage of transition, which indicates the significance of specific discharge education and post-discharge services. Moreover, the moderate overall readiness observed in this study also implies that a considerable number of patients were in the middle transitive stage between hospital reliance and independent self-management. Such results support the contribution of nurses in enhancing the preparedness of patients prior to discharge.

A critical point that should be taken into account when explaining these findings is the fact that perceived readiness and actual clinical readiness can be conceptually different. In this research, the aspect of readiness was measured by subjective views of the patients, which might not be consistent with objective clinical signs applied by the health workers. This distinction is especially relevant in the case of coronary artery bypass graft (CABG) surgery, when a patient with medical eligibility to leave the hospital can still feel insecure regarding the monitoring of his or her symptoms, medications, wound condition, physical activity, or lifestyle change. These perceived preparedness lapses might lead to an extended length of stay when a patient is medically stable, or the reverse, *where premature perceptions of preparedness doom a patient to post-discha*rge complications. Poor perceived preparedness can also have negative consequences on compliance with complicated post-CABG treatment protocols such as antiplatelet therapy, presence in cardiac rehabilitation, and self-management practices, which, in turn, can result in the occurrence of unwanted readmissions. The lack of objective clinical readiness indicators in this study makes it impossible to find out how differences between perceived and clinical readiness contribute to such outcomes. Future studies must consider combining the subjective and objective discharge readiness measures to better explain the combined effects on the length of stay, medication compliance, and readmission rates after CABG surgery. It should also be considered in future studies to determine the effectiveness of structured discharge education programs based on the literacy levels of patients and clinical requirements after CABG surgery. Moreover, exploring psychosocial factors (anxiety, depression, family support, and health literacy) can be used to understand further discharge readiness variability. Longitudinal studies are also required to determine the relationship between the readiness scores and the significant clinical outcomes, such as medication adherence, attendance at cardiac rehabilitation, and hospital readmission rates post-discharge. It should also be assessed in future studies to determine structured discharge education programs and health literacy-sensitive interventions and also to assess psychosocial determinants, including anxiety and family support, which might have an impact on discharge preparation following CABG surgery. Moreover, other potentially pertinent factors such as psychological condition, caregiver support, level of functional recovery, and content and quality of discharge education were not quantified in the current study, although other studies have indicated that such variables can play a role in discharge preparedness following cardiac surgery.

### 4.1 Limitations

This study has certain limitations that the results should be interpreted. First, the limited sample size of this study was due to one CABG surgery facility in Jordan and a stringent inclusion criterion. This limits the generalization of the findings to other groups such as urgent/emergent CABG, combined procedures, and other cultures or settings. Jordanian culture has its own family ties and community spirit of looking after ill family members, which may not be the same in other countries or cultures. Second, the cross-sectional design lacks causal inference, and it is necessary to take into consideration the possible confounding variables. Third, due to the absence of specific markers of clinical severity such as the number of grafts and postoperative or ICU complications, the disease’s complexity could not be properly controlled. Moreover, functional status and the level of family support were not directly measured in this study, although these two variables are known to affect discharge readiness after CABG surgery and may partly account for other variation not represented by the regression model. In addition, the regression model demonstrated a relatively small share of the discharge readiness variance (*R*^2^ = 0.15), indicating that there are additional clinical, psychological, and healthcare-related variables that were not used in this study and could also contribute to the discharge readiness of the patients. Fourth, because of a cross-sectional design, all the patients were evaluated at one discharge point. It has been mentioned that postoperative recovery of CABG surgery is highly variant regarding the presence of single patients, and they may have entirely different requirements. Furthermore, patients that undergo CABG surgery may not agree on a number of clinical variables that pertain to the severity of preoperative disease, comorbidity, and the complexity of the operation. The discussed variations that were not quantified in this study probably caused the heterogeneity in recovery status in the sample. This immeasurable variation has possibly been involved in the patients who have been ready to be discharged and affects the interpretation and generalization of the findings, but longitudinal studies are needed to demonstrate this relationship in the long run. Fifth, self-reporting surveys can also have caused a risk of response bias that can affect the responses and the findings. Sixth, psychological factors, e.g., anxiety, depression, and cognitive status, were not measured systematically, although they might have contributed to the sociodemographic variables and perceived readiness to leave the hospital. Seventh, years of formal education served as a proxy variable of health literacy, but health literacy itself, which is probably a more proximate determinant of discharge readiness, was not directly measured. Eighth, we failed to measure the content, time, and provider attributes of discharge education, and unmeasured variation in the quality or intensity of teaching might have influenced patient-reported readiness.

## 5 Conclusion

This study showed that patients undergoing coronary artery bypass graft (CABG) surgery reported a moderate level of readiness for hospital discharge on the RHDS, with particularly low scores in knowledge-related domains such as awareness of post-discharge complications and activity restrictions. Older age and lower educational level were identified as significant predictors of reduced discharge readiness, highlighting vulnerable patient groups who may require additional preparation before discharge. Interpreted through Meleis’s transition theory, these findings suggest that insufficient knowledge, limited coping capacity, and variable support may compromise the quality of the transition from hospital to home. These findings support the use of discharge readiness assessment and targeted education to facilitate a safe transition from hospital to home after CABG surgery.

## Data Availability

The raw data supporting the conclusions of this article will be made available by the authors, without undue reservation.

## Footnotes

1
https://www.marquette.edu/nursing/readiness-hospital-discharge-scale.php
